# Crystal structure of 1,3-bis­(2,6-diiso­propyl­phen­yl)-4,5-dimethyl-1*H*-imid­azol-3-ium bromide di­chloro­methane disolvate

**DOI:** 10.1107/S1600536814023150

**Published:** 2014-10-24

**Authors:** Matthias Berger, Norbert Auner, Michael Bolte

**Affiliations:** a Institut für Anorganische Chemie, J. W. Goethe-Universität Frankfurt, Max-von-Laue-Str. 7, 60438 Frankfurt/Main, Germany

**Keywords:** Arduengo-type carbene, C—H⋯Br hydrogen bond, crystal structure

## Abstract

The title solvated salt, C_29_H_41_N_2_^+^·Br^−^·2CH_2_Cl_2_ was obtained from the reaction of the Arduengo-type carbene 1,3-bis­(2,6-diiso­propyl­phen­yl)-1,3-dihydro-4,5-dimethyl-2*H*-imidazol-2-ylidene with Si_2_Br_6_ in di­chloro­methane. The complete cation is generated by a crystallographic mirror plane and the dihedral angle between the five-membered ring and the benzene ring is 89.8 (6)°; the dihedral angle between the benzene rings is 40.7 (2)°. The anion also lies on the mirror plane and both di­chloro­methane mol­ecules are disordered across the mirror plane over two equally occupied orientations. In the crystal, the cations are linked to the anions *via* C—H⋯Br hydrogen bonds.

## Related literature   

For the preparation of imidazolium salts, see: Arduengo *et al.* (1995 ▶, 1999 ▶); Hinter­mann *et al.* (2007 ▶); Gaillard *et al.* (2009 ▶). For silylene stabilization, see: Wang *et al.* (2008 ▶); Ghadwal *et al.* (2009 ▶); Filippou *et al.* (2009 ▶). For structures with the same cation but different anions, see: Clavier *et al.* (2009 ▶); Gaillard *et al.* (2009 ▶). For other crystallographically characterized imidazolium structures, see: Arduengo *et al.* (1995 ▶, 1999 ▶); Fliedel *et al.* (2007 ▶); Hagos *et al.* (2008 ▶); Berger, Auner & Bolte (2012 ▶); Berger, Auner, Sinke & Bolte (2012 ▶); Ikhile & Bala (2010 ▶); Giffin *et al.* (2010 ▶)
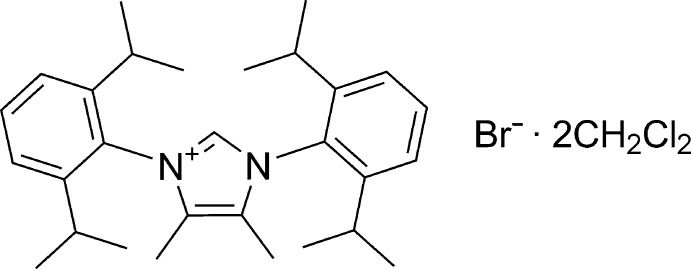


## Experimental   

### Crystal data  

C_29_H_41_N_2_^+^·Br^−^·2CH_2_Cl_2_
*M* *_r_* = 667.40
Monoclinic, 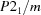

*a* = 10.0644 (11) Å
*b* = 16.6082 (17) Å
*c* = 10.7107 (15) Å
β = 98.48 (1)°
*V* = 1770.7 (4) Å^3^
*Z* = 2
Mo *K*α radiation
μ = 1.48 mm^−1^
*T* = 173 K
0.20 × 0.20 × 0.20 mm

### Data collection  

STOE IPDS II two-circle diffractometer
Absorption correction: multi-scan (*X-AREA* Stoe & Cie, 2001 ▶) *T*_min_ = 0.756, *T*_max_ = 0.756
21288 measured reflections
3229 independent reflections
2618 reflections with *I* > 2σ(*I*)
*R*_int_ = 0.156

### Refinement  

*R*[*F* ^2^ > 2σ(*F* ^2^)] = 0.163
*wR*(*F* ^2^) = 0.393
*S* = 1.12
3229 reflections
190 parameters
H-atom parameters constrained
Δρ_max_ = 1.09 e Å^−3^
Δρ_min_ = −1.13 e Å^−3^

### 

Data collection: *X-AREA* (Stoe & Cie, 2001 ▶); cell refinement: *X-AREA*; data reduction: *X-AREA*; program(s) used to solve structure: *SHELXS2013* (Sheldrick, 2008 ▶); program(s) used to refine structure: *SHELXL2013* (Sheldrick, 2008 ▶); molecular graphics: *XP* in *SHELXTL* (Sheldrick, 2008 ▶); software used to prepare material for publication: *SHELXL2013*, *PLATON* (Spek, 2009 ▶) and *publCIF* (Westrip, 2010 ▶).

## Supplementary Material

Crystal structure: contains datablock(s) I, global. DOI: 10.1107/S1600536814023150/hb7303sup1.cif

Structure factors: contains datablock(s) I. DOI: 10.1107/S1600536814023150/hb7303Isup2.hkl

Supporting information file. DOI: 10.1107/S1600536814023150/hb7303Isup3.cml

**Fig. 1.**
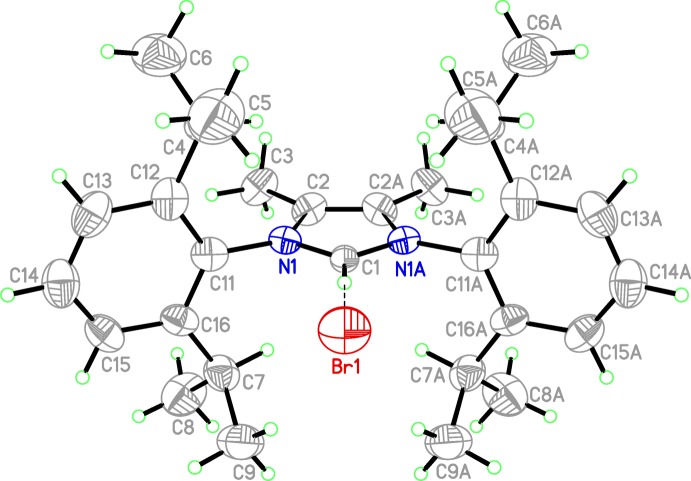
Perspective view of the title comopound with displacement ellipsoids drawn at the 50% probability level. The C—H···Br hydrogen bond is drawn as a dashed line. Atoms labelled with suffix A were generated by the symmetry operator x, -y + 1/2, z.

Click here for additional data file.x y z . DOI: 10.1107/S1600536814023150/hb7303fig1.tif
Perspective view of the title comopound with displacement ellipsoids drawn at the 50% probability level. The C—H⋯Br hydrogen bond is drawn as a dashed line. Atoms labelled with suffix A were generated by the symmetry operator *x*, −*y* + 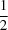, *z*.

CCDC reference: 1030231

Additional supporting information:  crystallographic information; 3D view; checkCIF report

## Figures and Tables

**Table 1:** Hydrogen-bond geometry (, )

*D*H*A*	*D*H	H*A*	*D* *A*	*D*H*A*
CHBr1	0.95	2.46	3.403(13)	172

## supplementary crystallographic information

### S1. Comment

Imidazolium salts are precursors for the synthesis of N-heterocyclic carbenes (NHC) and can be prepared according to Arduengo *et al.* (1995, 1999) and Hintermann (2007). To block deprotonation and substitution reactions at the unsaturated backbone of the imidazolium skeleton, methyl groups adjacent to the C=C bond can decrease NHC reactivity and increase the steric demand at these carbon positions (Gaillard *et al.*, 2009). Deprotonation of these imidazolium salts by strong bases gives the free stable NHC, which is widely used as a ligand for *e.g.* silylene stabilization (Wang *et al.*, 2008; Ghadwal *et al.*, 2009; Filippou *et al.*, 2009).

The title compound crystallizes with discrete cations, anions and solvent dichloromethane molecules. The cations and anions are located on a crystallographic mirror plane. Both dichloromethane molecules show a disorder across a mirror plane over two equally occupied positions. The Br anions are connnected to the cations *via* C—H···Br hydrogen bonds.

Structures with the same cation, but with different anions and solvent molecules, have been determined by Clavier *et al.* (2009) and Gaillard *et al.* (2009). For compounds with 1,3-Bis-(2,6-diisopropylphenyl)imidazolium unit, see: Ikhile *et al.* (2010), Giffin *et al.* (2010), Hagos *et al.* (2008), Fliedel *et al.* (2007), Berger, Auner & Bolte (2012); Berger, Auner, Sinke & Bolte (2012).

### S2. Experimental

The title compound is synthesized according to Arduengo *et al.* (1995), Hintermann (2007) and Gaillard *et al.* (2009).

1,3-Bis(2,6-diisopropylphenyl)-4,5-dimethyl-1*H*-imidazol-3-ium bromide chloroform disolvate was prepared by reacting 340 mg of 1,3-bis(2,6-diisopropylphenyl)-1,3-dihydro-4,5-dimethyl-2*H*- imidazol-2-ylidene with 300 mg of Si_2_Br_6_ in 10 ml dichloromethane. After removing the solvent *in vacuo* and dissolving the residue in CD_2_Cl_2_ the NMR-Tube was stored for two weeks at 253 K. Colourless needles of the title compound crystallized.

### S3. Refinement

All atoms have been anisotropically refined. H atoms were refined using a riding model, with C_aromatic_—H = 0.95 Å or C_methyl_—H = 0.98 Å, C—H_tertiary_ = 0.99 Å and with *U*_iso_(H) = 1.2*U*_eq_(C) or *U*_iso_(H) = 1.5*U*_eq_(C_methyl_).

### Crystal data

**Table:**

C_29_H_41_N_2_^+^·Br^−^·2CH_2_Cl_2_	*F*(000) = 696
*M**_r_* = 667.40	*D*_x_ = 1.252 Mg m^−^^3^
Monoclinic, *P*2_1_/*m*	Mo *K*α radiation, λ = 0.71073 Å
*a* = 10.0644 (11) Å	Cell parameters from 20226 reflections
*b* = 16.6082 (17) Å	θ = 3.2–25.8°
*c* = 10.7107 (15) Å	µ = 1.48 mm^−^^1^
β = 98.48 (1)°	*T* = 173 K
*V* = 1770.7 (4) Å^3^	Block, colourless
*Z* = 2	0.20 × 0.20 × 0.20 mm

### Data collection

**Table:**

STOE IPDS II two-circle diffractometer	2618 reflections with *I* > 2σ(*I*)
Radiation source: Genix 3D IµS microfocus X-ray source	*R*_int_ = 0.156
ω scans	θ_max_ = 25.0°, θ_min_ = 3.2°
Absorption correction: multi-scan (*X-AREA* Stoe & Cie, 2001)	*h* = −11→11
*T*_min_ = 0.756, *T*_max_ = 0.756	*k* = −19→19
21288 measured reflections	*l* = −12→12
3229 independent reflections	

### Refinement

**Table:**

Refinement on *F*^2^	0 restraints
Least-squares matrix: full	Hydrogen site location: mixed
*R*[*F*^2^ > 2σ(*F*^2^)] = 0.163	H-atom parameters constrained
*wR*(*F*^2^) = 0.393	*w* = 1/[σ^2^(*F*_o_^2^) + (0.1372*P*)^2^ + 27.7491*P*] where *P* = (*F*_o_^2^ + 2*F*_c_^2^)/3
*S* = 1.12	(Δ/σ)_max_ < 0.001
3229 reflections	Δρ_max_ = 1.09 e Å^−^^3^
190 parameters	Δρ_min_ = −1.13 e Å^−^^3^

### Special details

**Table:**

Experimental. ;
Geometry. All e.s.d.'s (except the e.s.d. in the dihedral angle between two l.s. planes) are estimated using the full covariance matrix. The cell e.s.d.'s are taken into account individually in the estimation of e.s.d.'s in distances, angles and torsion angles; correlations between e.s.d.'s in cell parameters are only used when they are defined by crystal symmetry. An approximate (isotropic) treatment of cell e.s.d.'s is used for estimating e.s.d.'s involving l.s. planes.
Refinement. ;

### Fractional atomic coordinates and isotropic or equivalent isotropic displacement parameters (Å^2^)

**Table:**

	*x*	*y*	*z*	*U*_iso_*/*U*_eq_	Occ. (<1)
N1	0.7876 (7)	0.1843 (5)	0.4157 (7)	0.0283 (17)	
C1	0.7093 (13)	0.2500	0.4193 (12)	0.026 (3)	
H1	0.6157	0.2500	0.4235	0.031*	
C2	0.9196 (9)	0.2098 (7)	0.4076 (9)	0.035 (2)	
C3	1.0285 (10)	0.1493 (7)	0.4038 (12)	0.046 (3)	
H3A	0.9910	0.0949	0.4060	0.069*	
H3B	1.0982	0.1569	0.4770	0.069*	
H3C	1.0678	0.1562	0.3261	0.069*	
C4	0.6880 (14)	0.1029 (8)	0.1817 (11)	0.054 (3)	
H4	0.7369	0.1553	0.1943	0.065*	
C5	0.5431 (19)	0.1214 (12)	0.1280 (16)	0.092 (6)	
H5A	0.5398	0.1470	0.0451	0.138*	
H5B	0.5049	0.1580	0.1852	0.138*	
H5C	0.4911	0.0714	0.1192	0.138*	
C6	0.7583 (18)	0.0522 (12)	0.0899 (14)	0.081 (5)	
H6A	0.8504	0.0401	0.1290	0.121*	
H6B	0.7602	0.0824	0.0117	0.121*	
H6C	0.7089	0.0017	0.0708	0.121*	
C7	0.7974 (12)	0.1111 (8)	0.6591 (11)	0.048 (3)	
H7	0.8410	0.1618	0.6351	0.057*	
C8	0.9077 (15)	0.0617 (10)	0.7486 (13)	0.069 (4)	
H8A	0.9385	0.0932	0.8247	0.103*	
H8B	0.9840	0.0504	0.7039	0.103*	
H8C	0.8689	0.0108	0.7723	0.103*	
C9	0.6840 (16)	0.1355 (10)	0.7316 (13)	0.068 (4)	
H9A	0.7215	0.1645	0.8085	0.102*	
H9B	0.6370	0.0873	0.7544	0.102*	
H9C	0.6206	0.1705	0.6785	0.102*	
C11	0.7393 (10)	0.1030 (7)	0.4224 (10)	0.037 (2)	
C12	0.6930 (11)	0.0638 (7)	0.3109 (11)	0.045 (3)	
C13	0.6476 (13)	−0.0157 (8)	0.3211 (13)	0.056 (3)	
H13	0.6160	−0.0452	0.2467	0.067*	
C14	0.6482 (13)	−0.0516 (8)	0.4379 (13)	0.057 (3)	
H14	0.6130	−0.1043	0.4434	0.068*	
C15	0.6993 (12)	−0.0112 (8)	0.5450 (12)	0.048 (3)	
H15	0.7018	−0.0373	0.6242	0.058*	
C16	0.7483 (10)	0.0679 (7)	0.5420 (10)	0.040 (2)	
Br1	0.36891 (18)	0.2500	0.4011 (2)	0.0582 (7)	
C1L	0.250 (3)	0.2500	0.711 (3)	0.19 (3)	
H1LA	0.2711	0.2180	0.6419	0.228*	0.5
H1LB	0.2390	0.3042	0.6806	0.228*	0.5
Cl1	0.3918 (6)	0.2500	0.8208 (6)	0.104 (3)	
Cl2	0.1100 (7)	0.2217 (5)	0.7384 (7)	0.074 (2)	0.5
C2L	1.165 (2)	0.2500	0.089 (2)	0.090 (9)	
H2LA	1.1999	0.2798	0.0233	0.108*	0.5
H2LB	1.2084	0.2646	0.1703	0.108*	0.5
Cl3	1.1536 (11)	0.1392 (7)	0.0594 (9)	0.100 (3)	0.5
Cl4	0.9934 (12)	0.2500	0.0734 (10)	0.253 (11)	

### Atomic displacement parameters (Å^2^)

**Table:**

	*U*^11^	*U*^22^	*U*^33^	*U*^12^	*U*^13^	*U*^23^
N1	0.022 (4)	0.037 (4)	0.025 (4)	−0.004 (3)	0.001 (3)	0.001 (3)
C1	0.023 (6)	0.028 (7)	0.027 (6)	0.000	0.005 (5)	0.000
C2	0.019 (4)	0.046 (5)	0.036 (5)	0.011 (4)	−0.004 (4)	−0.003 (4)
C3	0.024 (5)	0.055 (7)	0.058 (7)	0.003 (5)	0.005 (5)	−0.013 (6)
C4	0.069 (8)	0.055 (8)	0.035 (6)	−0.007 (6)	−0.003 (5)	−0.009 (5)
C5	0.107 (14)	0.091 (13)	0.071 (10)	0.020 (11)	−0.013 (9)	−0.002 (9)
C6	0.086 (11)	0.101 (13)	0.051 (8)	−0.012 (10)	−0.004 (7)	0.001 (8)
C7	0.051 (7)	0.046 (7)	0.046 (6)	0.008 (6)	0.005 (5)	0.010 (5)
C8	0.070 (9)	0.079 (10)	0.050 (8)	0.015 (8)	−0.015 (7)	−0.005 (7)
C9	0.081 (10)	0.076 (10)	0.053 (8)	0.008 (8)	0.026 (7)	0.007 (7)
C11	0.025 (5)	0.047 (6)	0.038 (5)	0.006 (4)	0.000 (4)	0.002 (5)
C12	0.032 (5)	0.044 (7)	0.055 (7)	0.004 (5)	−0.002 (5)	0.002 (5)
C13	0.050 (7)	0.054 (8)	0.061 (8)	0.002 (6)	−0.004 (6)	−0.015 (6)
C14	0.051 (7)	0.047 (7)	0.068 (8)	−0.003 (6)	−0.005 (6)	0.001 (6)
C15	0.045 (6)	0.051 (7)	0.049 (7)	0.003 (5)	0.007 (5)	0.015 (6)
C16	0.032 (5)	0.050 (7)	0.039 (6)	0.009 (5)	0.010 (4)	0.009 (5)
Br1	0.0341 (9)	0.0750 (13)	0.0665 (12)	0.000	0.0107 (7)	0.000
C1L	0.08 (2)	0.44 (8)	0.048 (15)	0.000	0.014 (14)	0.000
Cl1	0.044 (3)	0.183 (8)	0.079 (4)	0.000	−0.006 (3)	0.000
Cl2	0.055 (4)	0.097 (7)	0.068 (4)	−0.026 (3)	0.002 (3)	−0.002 (3)
C2L	0.057 (13)	0.17 (3)	0.047 (12)	0.000	0.010 (10)	0.000
Cl3	0.111 (7)	0.106 (7)	0.076 (6)	−0.021 (6)	−0.009 (5)	−0.006 (5)
Cl4	0.119 (8)	0.56 (3)	0.081 (6)	0.000	0.031 (6)	0.000

### Geometric parameters (Å, º)

**Table:**

N1—C1	1.350 (11)	C8—H8C	0.9800
N1—C2	1.409 (12)	C9—H9A	0.9800
N1—C11	1.440 (14)	C9—H9B	0.9800
C1—N1^i^	1.350 (11)	C9—H9C	0.9800
C1—H1	0.9500	C11—C12	1.380 (16)
C2—C2^i^	1.34 (2)	C11—C16	1.398 (15)
C2—C3	1.492 (14)	C12—C13	1.406 (18)
C3—H3A	0.9800	C13—C14	1.385 (19)
C3—H3B	0.9800	C13—H13	0.9500
C3—H3C	0.9800	C14—C15	1.362 (18)
C4—C5	1.52 (2)	C14—H14	0.9500
C4—C12	1.523 (17)	C15—C16	1.405 (17)
C4—C6	1.54 (2)	C15—H15	0.9500
C4—H4	1.0000	C1L—Cl2	1.56 (3)
C5—H5A	0.9800	C1L—Cl2^i^	1.56 (3)
C5—H5B	0.9800	C1L—Cl1	1.71 (3)
C5—H5C	0.9800	C1L—H1LA	0.9592
C6—H6A	0.9800	C1L—H1LB	0.9585
C6—H6B	0.9800	Cl2—Cl2^i^	0.941 (16)
C6—H6C	0.9800	C2L—Cl4	1.71 (3)
C7—C16	1.466 (17)	C2L—Cl3	1.867 (12)
C7—C9	1.527 (17)	C2L—Cl3^i^	1.867 (12)
C7—C8	1.585 (17)	C2L—H2LA	0.9648
C7—H7	1.0000	C2L—H2LB	0.9468
C8—H8A	0.9800	Cl3—Cl4	2.465 (14)
C8—H8B	0.9800	Cl4—Cl3^i^	2.465 (14)
			
C1—N1—C2	108.6 (8)	C7—C9—H9A	109.5
C1—N1—C11	123.6 (8)	C7—C9—H9B	109.5
C2—N1—C11	127.8 (8)	H9A—C9—H9B	109.5
N1^i^—C1—N1	107.9 (11)	C7—C9—H9C	109.5
N1^i^—C1—H1	126.1	H9A—C9—H9C	109.5
N1—C1—H1	126.1	H9B—C9—H9C	109.5
C2^i^—C2—N1	107.5 (5)	C12—C11—C16	124.3 (11)
C2^i^—C2—C3	132.4 (6)	C12—C11—N1	118.2 (9)
N1—C2—C3	120.2 (10)	C16—C11—N1	117.4 (9)
C2—C3—H3A	109.5	C11—C12—C13	116.6 (11)
C2—C3—H3B	109.5	C11—C12—C4	123.2 (11)
H3A—C3—H3B	109.5	C13—C12—C4	120.2 (11)
C2—C3—H3C	109.5	C14—C13—C12	121.1 (12)
H3A—C3—H3C	109.5	C14—C13—H13	119.5
H3B—C3—H3C	109.5	C12—C13—H13	119.5
C5—C4—C12	109.4 (12)	C15—C14—C13	119.9 (12)
C5—C4—C6	112.0 (12)	C15—C14—H14	120.0
C12—C4—C6	112.9 (12)	C13—C14—H14	120.0
C5—C4—H4	107.4	C14—C15—C16	122.1 (11)
C12—C4—H4	107.4	C14—C15—H15	118.9
C6—C4—H4	107.4	C16—C15—H15	118.9
C4—C5—H5A	109.5	C11—C16—C15	115.8 (11)
C4—C5—H5B	109.5	C11—C16—C7	123.2 (10)
H5A—C5—H5B	109.5	C15—C16—C7	120.9 (10)
C4—C5—H5C	109.5	Cl2—C1L—Cl1	123.9 (16)
H5A—C5—H5C	109.5	Cl2^i^—C1L—Cl1	123.9 (16)
H5B—C5—H5C	109.5	Cl2—C1L—H1LA	106.5
C4—C6—H6A	109.5	Cl2^i^—C1L—H1LA	128.3
C4—C6—H6B	109.5	Cl1—C1L—H1LA	106.1
H6A—C6—H6B	109.5	Cl2—C1L—H1LB	106.4
C4—C6—H6C	109.5	Cl1—C1L—H1LB	106.2
H6A—C6—H6C	109.5	H1LA—C1L—H1LB	106.8
H6B—C6—H6C	109.5	Cl2^i^—Cl2—C1L	72.4 (5)
C16—C7—C9	112.5 (11)	Cl4—C2L—Cl3	86.9 (8)
C16—C7—C8	112.4 (10)	Cl4—C2L—Cl3^i^	86.9 (8)
C9—C7—C8	109.9 (11)	Cl3—C2L—Cl3^i^	160.3 (15)
C16—C7—H7	107.3	Cl4—C2L—H2LA	113.4
C9—C7—H7	107.3	Cl3—C2L—H2LA	113.8
C8—C7—H7	107.3	Cl3^i^—C2L—H2LA	52.5
C7—C8—H8A	109.5	Cl4—C2L—H2LB	114.0
C7—C8—H8B	109.5	Cl3—C2L—H2LB	114.6
H8A—C8—H8B	109.5	Cl3^i^—C2L—H2LB	85.0
C7—C8—H8C	109.5	H2LA—C2L—H2LB	112.0
H8A—C8—H8C	109.5	Cl3^i^—Cl4—Cl3	96.5 (6)
H8B—C8—H8C	109.5		
			
C2—N1—C1—N1^i^	−1.0 (13)	C11—C12—C13—C14	0.8 (17)
C11—N1—C1—N1^i^	177.7 (6)	C4—C12—C13—C14	−178.3 (12)
C1—N1—C2—C2^i^	0.6 (8)	C12—C13—C14—C15	−3.1 (19)
C11—N1—C2—C2^i^	−178.0 (8)	C13—C14—C15—C16	2.1 (19)
C1—N1—C2—C3	179.2 (10)	C12—C11—C16—C15	−3.6 (15)
C11—N1—C2—C3	0.6 (15)	N1—C11—C16—C15	179.5 (9)
C1—N1—C11—C12	91.6 (12)	C12—C11—C16—C7	−179.8 (10)
C2—N1—C11—C12	−89.9 (12)	N1—C11—C16—C7	3.3 (15)
C1—N1—C11—C16	−91.3 (12)	C14—C15—C16—C11	1.1 (17)
C2—N1—C11—C16	87.2 (12)	C14—C15—C16—C7	177.4 (11)
C16—C11—C12—C13	2.6 (16)	C9—C7—C16—C11	104.4 (13)
N1—C11—C12—C13	179.5 (9)	C8—C7—C16—C11	−130.9 (12)
C16—C11—C12—C4	−178.2 (10)	C9—C7—C16—C15	−71.5 (14)
N1—C11—C12—C4	−1.3 (16)	C8—C7—C16—C15	53.1 (15)
C5—C4—C12—C11	−107.1 (14)	Cl1—C1L—Cl2—Cl2^i^	102.3 (9)
C6—C4—C12—C11	127.4 (13)	Cl3^i^—C2L—Cl3—Cl4	−72 (4)
C5—C4—C12—C13	72.0 (15)	Cl3—C2L—Cl4—Cl3^i^	−161.3 (14)
C6—C4—C12—C13	−53.5 (16)	Cl3^i^—C2L—Cl4—Cl3	161.3 (14)

Symmetry code: (i) *x*, −*y*+1/2, *z*.

### Hydrogen-bond geometry (Å, º)

**Table:**

*D*—H···*A*	*D*—H	H···*A*	*D*···*A*	*D*—H···*A*
C—H···Br1	0.95	2.46	3.403 (13)	172
