# Supplementary figures and images for: Crystal structure of 1,3-bis­(2,6-diiso­propyl­phen­yl)-4,5-dimethyl-1H-imid­azol-3-ium bromide di­chloro­methane disolvate
Source: Acta Crystallogr Sect E Struct Rep Online. 2014 Oct 24;70(Pt 11):o1193–4. doi: 10.1107/S1600536814023150 (PMC4257239; doi:10.1107/S1600536814023150)

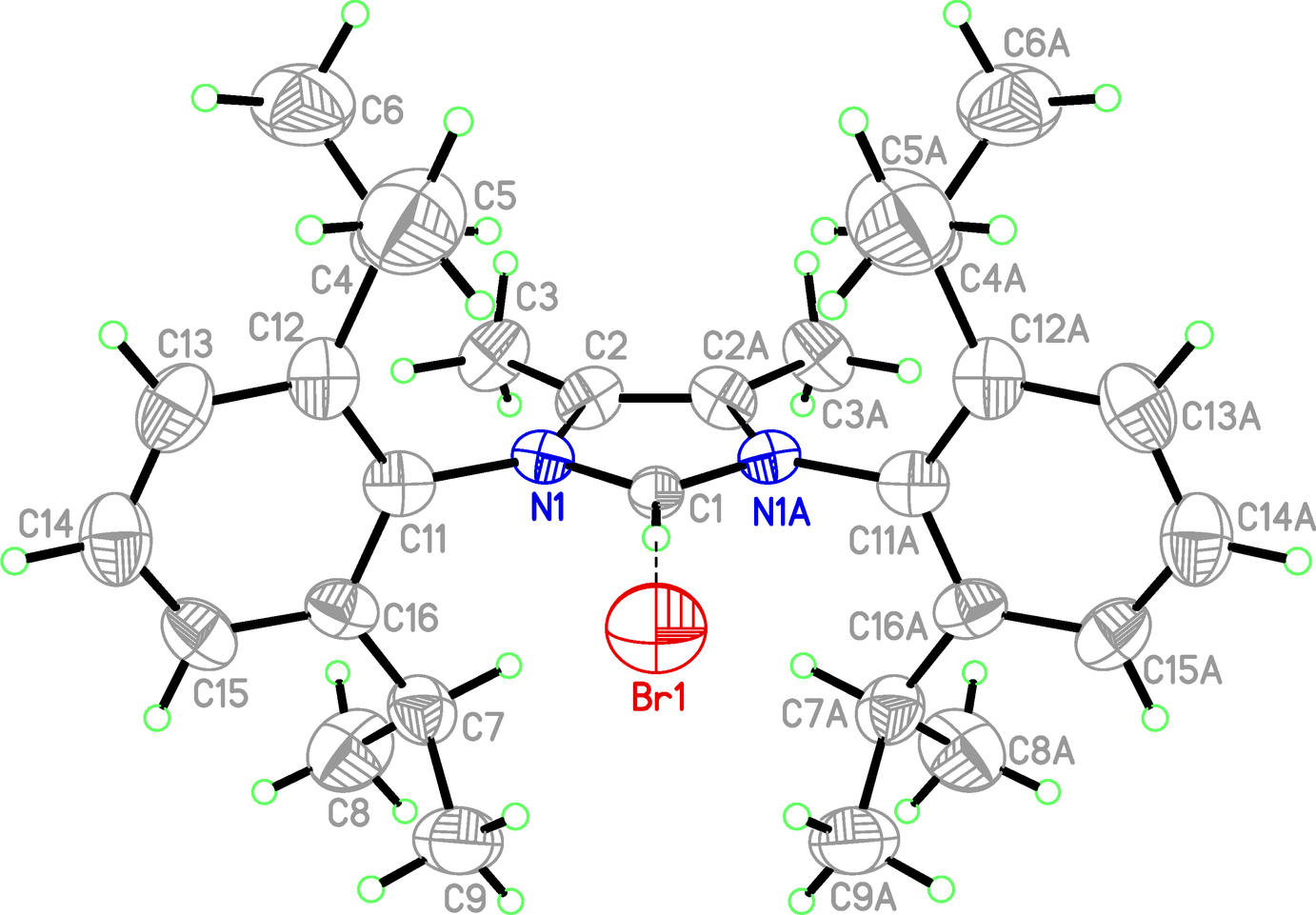

Supplement: Supplementary file 4 [file e-70-o1193-fig1.tif]
